# Response: Commentary: Using Directional Deep Brain Stimulation to Co-activate the Subthalamic Nucleus and Zona Incerta for Overlapping Essential Tremor/Parkinson's Disease Symptoms

**DOI:** 10.3389/fneur.2020.00100

**Published:** 2020-02-18

**Authors:** Drew Falconer, Sean Rogers, Mahesh Shenai

**Affiliations:** Inova Parkinson's and Movement Disorders Center, Falls Church, VA, United States

**Keywords:** directional deep brain stimulation, co-activating targets, single segment bipolar montage, Parkinson' disease, essential tremor (ET)

We have received the commentary by Wu et al. (1) of our case report (2) and welcome the discussion and alternative analysis. To summarize, we presented a patient with combined features of Parkinson's and Essential Tremor, both treated with a single unilateral subthalamic nucleus (STN) implant. The patient demonstrated symptomatic relief with single segment bipolar directional stimulation of segment 2B on the Abbott 6172 lead. We reasonably postulated that this dual effect of arresting both resting and action tremors resulted from the co-activation of the dorsolateral STN and the adjacent zona incerta (ZI) targets. The dual effect was only observed with single segment bipolar montage, whereas omnidirectional stimulation and monopolar directional stimulation resulted only in reducing rigidity and resting tremor. The commenters raise the concern that the electrode position was suboptimally placed on the lateral edge of the STN, straddling the internal capsule (IC), and therefore, segmental stimulation only activated the dorsolateral STN, achieving the traditional effect.

The commenters rely on our figure (Figure 3, Falconer et al.), to determine that the electrode was in a “suboptimal position.” We concur with the commenters that the figure can be interpreted in this manner, however we believe this perception is artifactual in nature. As the CT scan of the metal electrode causes artifact, biased in the direction of the electrode (anterolateral to posteromedial) due to slice averaging, there can be a perceived offset. Figure 1 shows this effect with low-threshold windowing in the axial (Figure 1A) and probe's eye (Figure 1B) views, compared to the respective high-threshold windows (Figures 1C,D). With higher-threshold windows, the centroid of the electrode cylinder converges well within the STN near the intended dorsolateral target, within 2.6 and 1.5 mm of the medial and lateral STN border, respectively (Figure 1E). Given the radius of the electrode is 0.7 mm, the 2B contact would be within 1.9 to 2.2 mm of the dorsomedial border of the STN, rather than ~5 mm as the commenters suggest.

**Figure 1:**
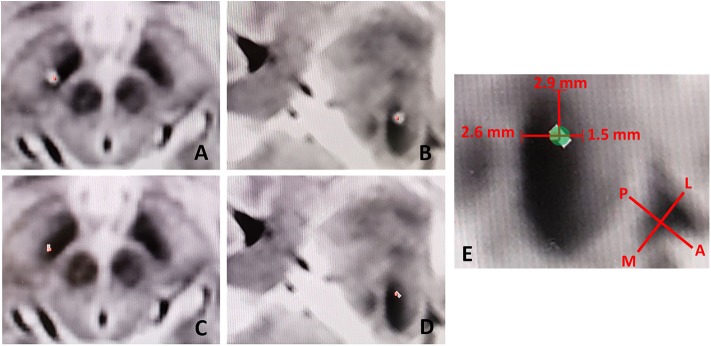
Low-threshold windowing in the axial **(A)** and probe's eye **(B)** views, compared to the respective high-threshold windows **(C,D)** showing reduction in electrode artifact and thus the perceived offset of the electrode. **(E)** Utilizing higher-threshold windows, the centroid of the electrode cylinder converges well within the STN near the intended dorsolateral target, within 2.6 and 2.9 mm of the medial and lateral STN border, respectively. L, Lateral; M, Medial; P, Posterior; A, Anterior.

During the case, detailed microelectrode recordings (MER) was performed, in addition to microstimulation and macrostimulation to assess side effects. The MER revealed 6 mm of STN with stereotypical STN patterns identified 4.5 mm above and to one 1.5 mm below target. This electrophysiologic length of the STN is consistent with a mid-STN trajectory, contrary to the commenter's suggestion. We routinely perform microstimulation through the MER electrode at the electrophysiological floor, middle and top of the STN. At the floor, we observed transient contralateral hand paresthesias above 2 mA without any capsular effects. Microstimulation at 3.5 mm above the target (which would be at the top of the 2B electrode) produced inconsistent and mild facial asymmetry at a relatively high 3 mA, and transient contralateral paresthesias below 2 mA. At the top of the STN, there were no side effects observed at any amplitude. Macrostimulation of all leads up to 4 mA did not produce any capsular side effects, as the commenters anticipated. Taken in all, this profile does not suggest a suboptimally placed electrode, but rather, a satisfactory placement. During macrostimulation, we achieved resting tremor and rigidity control with omnidirectional stimulation as well as monopolar directional stimulation. The kinetic tremor was controlled only with a single segment bipolar directional (2A+, 2B–, 2C+) montage. Based on this information, we can reasonably infer that only the single segment bipolar directional montage of activation was responsible for the dual therapeutic effect.

The commenters further ruminate that the inferred position of the electrode (~5 mm from the ZI) could not spatially activate the ZI based on modeling studies of volume tissue activation (VTA). Citing Madler and Coenen (3), they endorse the notion that 1.4 mA omnidirectional stimulation has a VTA of 2.5 mm. Based on our discussion above, we believe that the 2B contact is well within this range, between 1.8 and 2.1 mm away. Furthermore, the commenters endorse the notion that bipolar stimulation alone would not increase the VTA compared to monopolar stimulation based on Buehlmann et al. (4). We do concur with that statement, as our use of the 2B contact in a directional montage allowed us to increase stimulation amplitude without generating capsular effects in order to arrest the resting tremor. Nevertheless, even Buehlmann et. al. acknowledge in their discussion that the assumptions of their “simplified” model causes a “spatial underestimation” of VTA. Finally both studies (Madler et al. and Buehlmann et al.) consider models of the Medtronic Activa constant voltage stimulation, whereas our patient received Abbott's Infinity constant current stimulation. The VTA will be sensitive to assumptions of tissue impedances in any modeling and therefore literal application of a modeling study to an individual and real patient is a specious endeavor. If taken from a logical montage progression and functional programming response, the theory at the heart of this case report holds.

We therefore respectfully disagree with the commenter's conclusion that this case simply demonstrated salvage of a suboptimally placed electrode, as the above considerations of the radiographic artifact and electrophysiological record clearly endorse a reasonably placed electrode without observed capsular effects that the commenters anticipated. Nearly 2 years after surgery, this patient continues to have full mixed tremor control in the same single segment bipolar directional montage and has returned to playing the guitar professionally.

Ultimately, regardless of the underlying conflicting theories, we believe this case demonstrates that the use of a single segment bipolar directional montage can produce a differential and beneficial clinical effect possibly extending the area of stimulation to adjacent structures. The commenter's suggest an alternative theory that sole STN stimulation can sometimes impact both rest and kinetic tremor control, is certainly plausible. However, it has also been our experience that sole STN stimulation does not reliably control the kinetic tremor in these type of cases, and the functional response of this patient to localized STN stimulation did not yield kinetic tremor control. We hope we have succeeded in at least stimulating debate in this new and exciting era of directional neuromodulation.

## Author Contributions

MS and DF researched and wrote the response. SR edited the manuscript.

### Conflict of Interest

The authors declare that the research was conducted in the absence of any commercial or financial relationships that could be construed as a potential conflict of interest.
